# Anti-Obesity Effects of Microalgae

**DOI:** 10.3390/ijms21010041

**Published:** 2019-12-19

**Authors:** Saioa Gómez-Zorita, Jenifer Trepiana, Maitane González-Arceo, Leixuri Aguirre, Iñaki Milton-Laskibar, Marcela González, Itziar Eseberri, Alfredo Fernández-Quintela, María P. Portillo

**Affiliations:** 1 Nutrition and Obesity Group, Department of Nutrition and Food Science, University of the Basque Country (UPV/EHU) and Lucio Lascaray Research Institute, 01006 Vitoria, Spain; saioa.gomez@ehu.eus (S.G.-Z.); jenifer.trepiana@ehu.eus (J.T.); maitanega14@gmail.com (M.G.-A.); leixuri.aguirre@ehu.eus (L.A.); inaki.milton@ehu.eus (I.M.-L.); mariapuy.portillo@ehu.eus (M.P.P.); 2 CIBEROBN Physiopathology of Obesity and Nutrition, Institute of Health Carlos III, 01006 Vitoria, Spain; 3 Nutrition and Food Science Department, Faculty of Biochemistry and Biological Sciences, National University of Litoral and National Scientific and Technical Research Council (CONICET), Santa Fe 3000, Argentina; maidagon@fbcb.unl.edu.ar

**Keywords:** microalgae, obesity, triglyceride, adipose tissue, adipocyte, mice

## Abstract

In recent years, microalgae have attracted great interest for their potential applications in nutraceutical and pharmaceutical industry as an interesting source of bioactive medicinal products and food ingredients with anti-oxidant, anti-inflammatory, anti-cancer, and anti-microbial properties. One potential application for bioactive microalgae compounds is obesity treatment. This review gathers together in vitro and in vivo studies which address the anti-obesity effects of microalgae extracts. The scientific literature supplies evidence supporting an anti-obesity effect of several microalgae: *Euglena gracilis*, *Phaeodactylum tricornutum*, *Spirulina maxima*, *Spirulina platensis*, or *Nitzschia laevis*. Regarding the mechanisms of action, microalgae can inhibit pre-adipocyte differentiation and reduce de novo lipogenesis and triglyceride (TG) assembly, thus limiting TG accumulation. Increased lipolysis and fatty acid oxidation can also be observed. Finally, microalgae can induce increased energy expenditure via thermogenesis activation in brown adipose tissue, and browning in white adipose tissue. Along with the reduction in body fat accumulation, other hallmarks of individuals with obesity, such as enhanced plasma lipid levels, insulin resistance, diabetes, or systemic low-grade inflammation are also improved by microalgae treatment. Not only the anti-obesity effect of microalgae but also the improvement of several comorbidities, previously observed in preclinical studies, has been confirmed in clinical trials.

## 1. Introduction

Microalgae are prokaryotic or eukaryotic microscopic single-cell organisms, found in fresh water and marine systems. They produce approximately half of the atmospheric oxygen and use the greenhouse gas carbon dioxide to grow photo-autotrophically. Together with bacteria, microalgae provide energy for all the trophic levels above them. Although microalgae show a great biodiversity, the ones most studied are *Chlorella, Spirulina, Haematococus, Dunaniella* y *Scenedesmus*.

Microalgae produce a great variety of compounds, such as photosynthetic pigments (carotenoids and chlorophylls), sterols, polyunsaturated fatty acids, vitamins, minerals, fiber, polysaccaharides, enzymes, peptides, and toxins. It is important to emphasize that the chemical composition of microalgae depends on the species and the cultivation conditions, such as temperature, illumination, pH, CO_2_ supply, salt, and nutrients [1,2]. They have attracted great interest in recent years due to their potential applications in nutraceutical and pharmaceutical industries, and are a major source of bioactive medicinal products and food ingredients with anti-oxidant, anti-inflammatory, anti-cancer, and anti-microbial properties [2,3].

One of the potential application fields for the microalgae bioactive compounds is obesity, which has become a serious health problem due to its high prevalence, and because it is a major risk factor for a wide range of chronic diseases, including diabetes, cardiovascular diseases, and cancer [4,5,6]. Nowadays, approved new-generation anti-obesity medications offer a safe and tolerable adjunct to lifestyle interventions for the majority of individuals with obesity. Nevertheless, depending on patient tolerability to side effects, poor adherence or discontinuation can be treatment limitations. In fact, this situation reduces treatment benefits [7].

In this context, the present review gathers in vitro and in vivo studies addressed to analyze the anti-obesity effects of microalgae extracts, but not those where isolated microalgae compounds have been used.

## 2. In Vitro Studies

**Figure 1:**
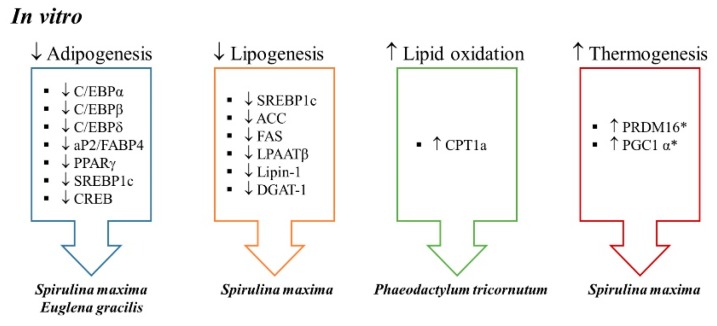
Anti-obesity mechanisms of action described in in vitro studies (* ex vivo). ACC: acetyl-CoA carboxylase, AP2: fatty acid binding protein, C/EBP: CCAAT-enhancer-binding protein, CPT1: carnitine palmitoyltransferase 1, CREB: cAMP regulatory element-binding protein; DGAT-1: diacylglycerol O-acyltransferase, FABP4: fatty acid-binding protein 4, FAS: fatty acid synthase, LPAATβ: lysophosphatidic acid acyltransferase β, PGC-1α: peroxisome proliferator-activated receptor gamma co-activator 1α, PRDM16: PR domain-containing 16, PPARγ: peroxisome proliferator activated receptor γ, SREBP1c: sterol regulatory element-binding protein 1c. ↑ significant increase, ↓: significant decrease.

To date, several in vitro studies have been conducted to analyze the effects of microalgae extracts on adipogenesis and metabolic processes involved in triglyceride (TG) accumulation (Table 1; Figure 1).

Sugimoto et al. [8] used an aqueous extract of *Euglena gracilis Z* (Euglena), unicellular photosynthesizing green algae. Euglena contains vitamins, minerals, unsaturated fatty acids, and accumulates crystalline β-1,3-glucan, a polysaccharide also known as paramylon, which is considered a functional dietary fiber. The authors obtained human adipose-derived stem cells (hASCs) from a non-diabetic female donor with a body mass index (BMI) of 26 kg/m^2^, and differentiated these cells into adipocytes for 7 days (0–7 days). Cells were cultured for an additional 7-day maturing period (8–14 days). Cytotoxicity was not observed in cells treated with any of the checked Euglena extract dilutions used (1.25%, 2.5%, 5%, 10%, 20%, or 40%). When the lipid content of cells incubated with the extract at doses of 5%, 10%, or 20% was analyzed, the authors observed that Euglena extract reduced cellular TG content 17%, 44%, and 74%, in line with the increased concentration of the extract in the medium.

In order to explore the mechanism underlying this effect, the authors studied the adipogenic pathway. Adipogenesis is a tightly regulated cellular differentiation process, which allows adipose tissue expansion. In this process, mesenchymal stem cells become pre-adipocytes and pre-adipocytes differentiate into mature adipocytes, the cells that are able to accumulate triglycerides into lipid droplets [12]. For this purpose, they measured gene and protein expressions of peroxisome proliferator-activated receptor γ (PPARγ) and CCAAT-enhancer-binding protein α (C/EBPα), the master regulators of adipocyte-differentiation. While the gene expression of *Pparγ* and *C/ebpα* were increased during adipocyte-differentiation in the control cells, these were repressed by 23% when 20% of Euglena extract was added to the medium. Protein amounts of PPARγ and C/EBPα were also significantly reduced, which is consistent with this result. The authors also observed an inhibition induced by the Euglena extract in gene expression of adipogenic markers expressed downstream in the adipocyte differentiation process, and regulated by PPARγ and C/EBPα, such as fatty acid binding protein (*Ap2*) (also known as fatty acid bonding protein 4, *Fabp4*) and lipoprotein lipase (*Lpl*). These results show that Euglena extract inhibits adipocyte-differentiation through suppression of master regulators involved in that metabolic pathway.

Furthermore, since *Pparγ* expression is enhanced at the early phase of adipocyte-differentiation by two members of the C/EBP protein family, C/EBPβ and C/EBPδ, as well as by sterol regulatory element-binding transcription factor 1c (SREBP1c) and cAMP regulatory element-binding protein (CREB), their gene expression was also determined in hASCs. For this purpose, cells were cultured with or without Euglena extract (20%) during the first three days in the differentiation process. All these genes were downregulated when Euglena extract was present in the medium, showing that its inhibitory effect on adipocyte-differentiation was caused by repressing the early stage of adipocyte-differentiation. These observations were confirmed when adipogenesis was evaluated by determining Oil Red O from cells treated with 20% of extract during adipocyte-differentiation (days 0–7) and from those cells treated during adipocyte maturation (days 8–14). Constant supplementation (day 0–14) with Euglena extract inhibited lipid accumulation by approximately 50% as compared to the control cells. When supplementation with the alga extract took place only during the adipocyte differentiation period (days 0–7) lipid accumulation was inhibited by 60%, but approximately 96% of the accumulated lipids remained in the cells treated with the extract on days 7–14. Therefore, the authors concluded that Euglena extract suppresses adipocyte-differentiation at the early stage, thus contributing to its anti-obesity effect.

Another microalga studied by several authors is *Spirulina maxima*. It contains pigment proteins such as chlorophyll a and C-phycocyanin, which have been reported as possessing anti-oxidant, anti-inflammatory (both pigments), and anti-diabetic actions (C-phycocyanin). Seo et al. [9] performed an in vitro study to explore whether an ethanolic extract of this microalga also showed anti-obesity and adipocyte browning properties. For this purpose, 3T3-L1 pre-adipocytes and C3H10T1/2 cells, a cellular line functionally similar to mesenchymal stem cells, were treated during the differentiation period (0–8 days) with 50 or 100 µg/mL of the microalga extract. Previously no cytotoxicity had been confirmed at these concentrations. In 3T3-L1 pre-adipocytes, the addition of the extract to the differentiation medium decreased TG accumulation in a dose dependent manner. This effect was due to lower protein expression of the adipogenic regulators C/EBPα, PPARγ, and aP2, meaning that adipogenesis was inhibited. The same results were observed when the authors treated C3H10T1/2 cells with the extract at a concentration of 100 µg/mL.

In addition, the authors explored lipogenesis, the metabolic process through which fatty acids are esterified with glycerol for their storage as triglycerides, and that allows adipocytes to increase their size. More specifically, the authors measured enzymes involved in de novo lipogenesis, the biosynthetic pathway by which acetyl-CoA is converted to fatty acids before they are esterified with glycerol to synthesize triglycerides. For that purpose, authors measured proteins such as acyl-CoA carboxylase (ACC) and fatty acid synthase (FAS), as well as markers involved in triglyceride assembly, such as lysophosphatidic acid acyltransferase β (LPAATβ), lipin-1 and diacylglycerol acyltransferase-1 (DGAT1). In this regard, they observed that treating 3T3-L1 pre-adipocytes during differentiation with the ethanolic extract led to reductions in the protein expressions of SREBP1, ACC, and FAS, as well as of LPAATβ, lipin-1, and DGAT1. As far as C3H10T1/2 cells are concerned, incubating cells with the extract obtained from *Spirulina maxima* during differentiation reduced protein expressions of the three latter lipogenic markers (LPAATβ, lipin-1, and DGAT1). The authors concluded that this extract significantly suppressed lipogenesis either in 3T3-L1 adipocytes or C3H10T1/2 cells. Finally, the authors reported browning effects ex vivo, in cells obtained from the stromal vascular fraction of mice fed a high-fat diet (HFD) supplemented with an ethanolic extract of the alga (150 or 450 mg/kg/day). Cells were differentiated into white adipocytes, and higher protein expression of PR domain containing 16 (PRDM16) and uncoupling protein 1 (UCP1) was detected in the adipose primary cells. The expression of peroxisome proliferator-activated receptor gamma coactivator 1-alpha (PGC1α) was upregulated only by the higher dose.

Using *Phaeodactylum tricornutum*, a diatom microalga rich in eicosapentanoic acid (EPA) and the carotenoid fucoxanthin, Koo et al. [10] aimed to evaluate the anti-obesity effect of a commercially available extract, containing 3.5–6% fucoxanthin (*w*/*w*), on lipid accumulation in 3T3-L1 adipocytes. The cells were cultured during the differentiation period for six days with the *Phaeodactylum tricornutum* extract (100, 125, 200, 250, and 400 µg/mL), fucoxanthin (active principle; 10, 20, and 40 µg/mL) or curcumin as control (20 µg/mL). The microalga extract reduced adipogenesis in 3T3-L1 preadipocytes at a concentration of 250 µg/mL, and consequently reduced cellular lipid accumulation was observed. When looking at the mechanisms underlying this effect, the authors reported that although no changes were observed in the protein expression of the adipogenic factor C/EBPα, *Phaeodactylum tricornutum* extract decreased PPARγ protein expression and increased that of UCP1, mainly at the highest dose (400 µg/mL). Therefore, the authors concluded that *Phaeodactylum tricornutum* extract exhibits anti-obesity effects by controlling lipid metabolism through PPARγ and UCP1.

Finally, Gille et al. [11] incubated 3T3-L1 cells on day 7 of differentiation with an ethanolic extract of the same microalga, at a dose of 100 mg/L for 24 h. Moreover, they also tested its bioactive compound fucoxanthin at a concentration of 5 μM. According to the authors, each 100 mg/L of the microalga contained 3.6 μM of fucoxanthin. Regarding the microalga effect, the authors did not appreciate significant effects on lipid content or cell toxicity, although cluster of differentiation 36 (*Cd36*) and carnitine palmitoyltransferase 1a (*Cpt1a*) mRNA levels were significantly increased. Furthermore, *Cpt1a* gene expression was similarly induced by fucoxanthin incubation. Consequently, it can be proposed that the effect of the microalga extract on *Cpt1a* expression were due, at least in part, to its fucoxanthin content.

## 3. Animal Studies

**Figure 2:**
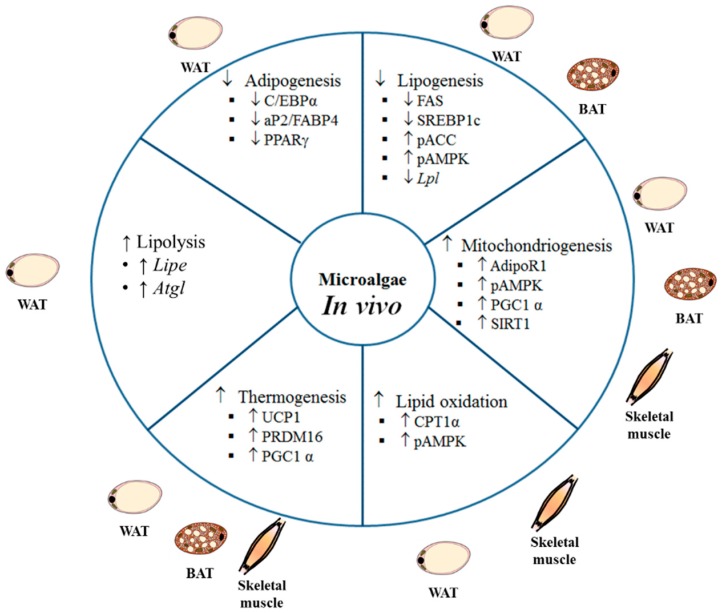
Anti-obesity mechanisms of action described in in vivo studies. pACC: phosphorylated acetyl-CoA carboxylase, AdipoR1: adiponectin receptor 1, pAMPK: phosphorylated AMP-activated protein kinase, AP2: fatty acid binding protein, BAT: brown adipose tissue, C/EBPα: CCAAT-enhancer-binding protein α, CPT: carnitine palmitoyltransferase, FABP: fatty acid binding protein, FAS: fatty acid synthase, LIPE: hormone sensitive lipase, LPL: lipoprotein lipase, PGC-1α: peroxisome proliferator-activated receptor gamma co-activator 1α, PPARγ: peroxisome proliferator activated receptor γ, PGC1α: PPARG coactivator 1 alpha, PRDM16: PR domain-containing 16, SIRT1: sirtuin 1, SREBP1c: sterol regulatory element-binding protein 1c, UCP1: uncoupling protein 1, WAT: white adipose tissue. ↑ significant increase, ↓: significant decrease.

Studies using animal models and different experimental approaches to analyze the potential anti-obesity effect of microalgae have revealed beneficial effects on body weight management and energy metabolism (Table 2; Figure 2). 

Seo et al. [9] carried out a study in Male Institute of Cancer Research (ICR) mice fed either a standard diet (SD; 18% of energy from fat) or a high-fat diet (HFD; 60% of energy from fat), supplemented or not with a commercially available ethanolic extract of *Spirulina maxima* at doses of 150 or 450 mg/kg body weight/day, for 6 weeks. Supplementation of the HFD with the microalga extract led to a significant reduction both in body weight gain as well as in subcutaneous and visceral adipose tissues, but a dose-dependent response was not observed. In addition, after the treatment, decreases in fasting glucose, TG, total cholesterol (TC), and low-density lipoprotein cholesterol (LDL-cholesterol), as well as increases in high-density lipoprotein cholesterol (HDL-cholesterol), were observed. 

By exploring the mechanisms responsible for the anti-obesity of the microalga extract, the authors observed lower protein expressions of adipogenesis and browning markers in white adipose tissue of mice fed the HFD supplemented with the extract. Thus, the authors detected a decrease in protein expression of C/EBPα, PPARγ, and aP2, with both extract doses. They also found increased expression of proteins related to thermogenesis, such as PRDM16 and PGC1α, not only in brown but also in white adipose tissue. These results suggest that the extract obtained from *Spirulina maxima* ameliorated the obesity induced by HFD by decreasing adipogenesis and increasing energy expenditure via thermogenesis.

Heo et al. [13] also tested the effects of *Spirulina maxima,* but in this case in rats. In their study, Sprague Dawley rats were fed a standard-fat diet (LFD group; 10% of energy from fat) or a HFD (60% of energy from fat) for 6 weeks. After this period, rats previously fed the HFD were divided into four groups: rats fed the same diet (HFD group), rats fed the HFD supplemented with an extract of *Spirulina maxima* at a dose of 62.5 mg/kg body weight/day (*Spirulina maxima* 62.5), rats fed the HFD supplemented with an extract of *Spirulina maxima* at a dose of 125 mg/kg body weight/day (*Spirulina maxima* 125) and rats fed the HFD supplemented with an extract of *Spirulina maxima* at a dose of 250 mg/kg body weight/day (*Spirulina maxima* 250). The cultivated microalgae were harvested by centrifugation (with a tubular separator), stored at –50 °C and lyophilized. All *Spirulina maxima* samples were administered orally for 4 weeks after being dissolved in carboxymethyl cellulose.

The body weight increase induced by high-fat feeding, as well as the increase in white adipose tissue, were significantly reduced after supplementation with *Spirulina maxima* in a dose-dependent manner. Haemotoxylin and eosin staining of epididymal adipose tissue revealed that the treatment reduced the increase in adipocyte size induced by the HFD at all the tested doses. With regard to brown adipose tissue index, no change was found in the HFD group when compared to the LFD group, but all the treatments with *Spirulina maxima* induced a significant increase in this parameter. The biochemical analysis revealed that the supplementation with *Spirulina maxima* attenuated a diet-induced decrease in adiponectin and increase of leptin and tumor necrosis factor α (TNF-α). It is well-known that obesity is commonly accompanied by insulin resistance, and for this reason serum glucose and insulin levels were also measured, and the Homeostatic Model Assessment for Insulin Resistance (HOMA-IR) was calculated. The *Spirulina maxima* 250 group showed reduced glucose and insulin levels (insulin levels also in the *Spirulina maxima* 125 group), and all the tested doses of the microalga diminished HOMA-IR values, reflecting the amelioration in insulin resistance induced by the HFD. In addition, TC was reduced in rats treated with 125 and 250 mg/kg body weight/day of *Spirulina maxima*, and the HDL-c/TC ratio was increased at all the tested doses. Furthermore, other parameters were measured in order to estimate the potential toxicity of microalga extract in the liver and kidneys, but all values were found in normal ranges. In the case of alanine aminotransferase (ALT), all doses reduced the increase induced by the HFD.

In order to gain insight into the molecular mechanisms underlying the observed effects, some gene and protein expressions were measured in epididymal adipose tissue and skeletal muscle. In adipose tissue, the HFD reduced the activated form of 5’ AMP-activated protein kinase (pAMPK), which leads to a decreased expression of the phosphorylated form of ACC, which is the inactive form of this enzyme, and increased protein expression of SREBP1 and FAS. The addition of the two highest doses of *Spirulina maxima* to the diet prevented both these effects as well as the increase in gene expression of *Srebp1* and *Fasn*. As far as the lipolytic and oxidative pathways are concerned, HFD decreased gene expression of adipose triglyceride lipase (*Atgl*) and *Cpt1*, whereas that of nuclear factor κΒ (*NfκΒ*) was increased. Those changes were prevented by *Spirulina maxima* supplementation (*Atgl* and *Cpt1* with the highest dose and *NfκΒ* with the two highest doses). As in the case of adipose tissue, the highest two doses of *Spirulina maxima* activated AMPK in skeletal muscle, and increased *Cpt1* and *Ucp2* gene expressions. A similar pattern of response was observed in both adipose tissue and skeletal muscle for adiponectin receptor (AdipoR1) in that gene and protein expressions increased in the *Spirulina maxima* 125 and *Spirulina maxima* 250 groups. Finally, when the authors analyzed the protein expression of nicotinamide phosphoribosyltransferase (NAMPT) and sirtuin 1 (SIRT1), they observed reduced values in both proteins either in adipose tissue and skeletal muscle from rats fed the HFD, that were prevented by *Spirulina maxima* treatment.

Another microalga studied for its anti-obesity properties is *Phaeodactylum tricornutum*. Gille et al. [11] analyzed the effects of this diatom microalga in adipose tissue in mice fed a HFD. For this purpose, male C75BL/6J mice were divided into three experimental groups: the HFD group fed a diet that provided 45% kcal from fat (mainly lard), the PE100 group fed the same diet supplemented with an ethanolic extract of the microalga, at a dose of 100 mg/kg body weight/day, and the PE300 group fed the same diet supplemented with an extract of the microalga, at a dose of 300 mg/kg body weight/day. Animals were maintained under these experimental conditions for 26 days. At the end of the experimental period, body weight gain, adipose depot weight, adipocyte size distribution, and the expression of lipid and energy metabolism-related genes were analyzed in adipose tissue. Although the energy intake was similar in the three groups, the PE300 group gained less body weight and showed less total body fat mass than the HFD group did. Body weight lost over a 6-h fasting period (an indicator of energy expenditure) was higher in the PE300 group. Regarding the fat depots, in PE300 group epididymal and inguinal tissues decreased by 24% and 17% respectively, compared to control mice and the PE lowest concentration was without effect. In addition, gene expression of mesoderm-specific transcript homolog protein (*Mest*), a marker of white adipose tissue expansion, was reduced in inguinal white adipose tissue of mice supplemented with the highest dose of the microalga extract. The authors also found that inguinal white adipose tissue of mice from both PE groups contained higher percentage of smaller adipocytes and lower percentage of large ones. Moreover, in this tissue, crown-like structures (CLS, microscopic foci of dying adipocytes surrounded by macrophages), which were positive for immunostaining against the macrophage marker galectin-3 (MAC-2), were found in all the animals of the PE300 group. Regarding plasma parameters, there was a tendency towards reduced HOMA-IR index only in the group supplemented with PE100, due to the significant decrease in fasting glucose. Moreover, the authors observed that fucoxanthin metabolites, such as fucoxanthinol and amarouciaxanthin were found in interscapular brown adipose tissue, epididymal and inguinal white adipose tissues from mice treated with the microalga extracts.

In order to analyze fatty acid metabolism, the expression of several related genes was measured in epididymal and inguinal white adipose tissues. In epididymal depot hormone sensitive lipase (*Hsl)*, *Perilipin 1* (*Plin1*), and *Lpl* genes were downregulated, and in inguinal WAT *Ucp1* and *Cpt1* were upregulated in the PE300 group, indicating higher fatty acid oxidation and thermogenesis in this group. The authors also analyzed brown adipose tissue. The activation of this tissue in PE groups was proposed based on the smaller size of brown adipocytes and the enrichment in UCP1 protein, measured by immunostaining, which was confirmed by Western blot. Finally, brown adipose tissue gene expression of *Cd36* and *Ppargc1a* was also increased in the PE100 group; whereas the lipolytic gene *Hsl* (both doses), and the lipogenic gene *Fasn* (both doses) underwent downregulation.

This microalga was also studied by Koo et al. [10] using a commercially available extract containing 3.5–6% fucoxanthin (*w*/*w*). In this study, which included an in vitro approach previously described in this review, female C57BL/6J mice were divided into six experimental groups. Thus, mice were fed a normal diet (ND), a HFD, or a HFD supplemented with the extract at a dose of 0.81 mg/kg body weight/day (PE-L), 1.62 mg/kg body weight/day (PE-M), or 3.25 mg/kg body weight/day (PE-H). After 6 weeks of treatment, the area under the curve (AUC) of body weights was higher in the HFD group than in the other experimental groups. *Phaeodactylum tricornutum* extract reduced total fat volume (measured by Micro Computed Tomography Analysis) (at all doses), abdominal adipose tissues (all doses), and subcutaneous depots (PE-M and PE-H groups). When looking at the mechanisms underlying these effects, the authors observed decreased protein expression of the adipogenic factors C/EBPα and PPARγ and increased protein expression of UCP1. Changes in the expressions of PPARγ and UCP1 had been observed in the in vitro experiments performed by these authors (Koo et al. [10]). This fact, linked with the changes to C/EBPα protein expression observed in vivo, suggests that *Phaeodactylum tricornutum* extract could activate thermogenesis and inhibit adipogenesis. Moreover, regarding plasma lipid profile, some changes were observed in animals treated with the *Phaeodactylum tricornutum* extract. Thus, while it decreases TG plasma levels at the medium dose (PE-M group), when it was added at a high dose (PE-H group) a decrease in LDL-cholesterol fraction was reported.

In addition to these studies, Kim et al. [16] studied the effect of the same microalgae in C57BL/6 mice. They observed that neither body weight increase, nor food intake were changed along the treatment period, although perirrenal and epididymal adipose depot weights were significantly decreased by the *Phaeodactylum tricornutum* extract. Due to the fact that these are the only data concerning anti-obesity effects, a longer description was not carried out and it has not been included in the pertinent table.

Sakanoi et al. [14] studied the beneficial effects of a spray-dried *Euglena gracilis* extract on mice. For this purpose, male C57BL/6J mice were fed a high-sucrose diet supplemented or not with *Euglena gracilis* extract (1%), for 8 weeks. At the end of the experimental period, no changes were observed in body weight or food intake. By contrast, total adipose tissue, perinephric and epididymal fat depots were reduced by the extract. mRNA levels of genes related to fatty acid synthesis (*Fasn,* glucose-6-phosphate dehydrogenase (*G6pdh*), malic enzyme (*Me*) and *Srebf1*), adipogenesis (*Pparγ*), and lypolisis (*Hsl*) were studied. The only change promoted by the microalga extract was an increase in *Hsl* gene expression. Dyslipidemia is another well-known complication of obesity but under these experimental conditions, serum lipids (TG, cholesterol and phospholipids) were not regulated by *Euglena gracilis*.

In a recent study conducted by Guo et al. [15], the effect of an extract of *Nitzschia laevis*, a diatom microalga, was studied. For this purpose, C57BL/6J mice were divided into four experimental groups: the ND group was fed a normal chow diet (4.1% of energy from fat), the HFD group was fed a high-fat diet (24% of energy from fat), the HFD-LE group was fed the high-fat diet supplemented with a low-dose of *Nitzschia laevis* (10 mg/kg body weight/day), and the HFD-HE group was fed the high-fat diet supplemented with a high-dose of *Nitzschia laevis* (50 mg/kg body weight/day). The *Nitzschia laevis* was administered daily by oral gavage for 8 weeks.

At the end of the experimental period, greater body weights were found in the three HFD-fed groups when compared to the ND group. Of these, the group supplemented with the highest dose of *Nitzschia laevis* showed lower body weight than that observed in the HFD group. The food intake records confirmed that the body weight-lowering effect was not due to a reduction in food consumption. When the weights of different white adipose tissue depots were analyzed, significantly lower values were appreciated in the epididymal fat depot of both *Nitzschia laevis*-supplemented groups, without differences between them. A similar pattern was observed in the diameters of the adipocytes of this adipose depot.

As far as brown adipose tissue is concerned, weight was significantly greater in the groups fed the HFD, when compared to the ND group, with no differences among them. By contrast, significantly decreased adipocyte numbers were appreciated in these same groups when compared to the ND group, suggesting that the seaweed supplementation had an adipocyte hypertrophy attenuating effect on these animals. Among the HFD-fed groups, those receiving the microalga supplementation showed increased adipocyte numbers when compared with the HFD group, which reached the ND group values in the case of the group treated with the lower dose. In order to gain a better understanding of the mechanisms that may be involved in the observed effects, gene expression of *Ucp1* and *Pgc-1α* was measured in this tissue. *Ucp1* was upregulated in the two *Nitzschia laevis*-supplemented groups when compared to both the ND and the HFD groups. In the case of *Pgc-1α* gene expression, an increase was only appreciated in the HFD-LE group, when compared to the other groups. Based on the results reported by the authors, a potential involvement of ucp-1 mediated thermogenesis in the body weight lowering effect observed in the *Nitzschia laevis*-supplemented groups cannot be ruled out.

In this study, the authors also analyzed the effects of *Nitzschia laevis* in gut epithelium integrity and gut microbiota. In this regard, they found that HFD feeding significantly decreased the expression of occludin, a plasma membrane protein considered as an important biomarker of the integrity and barrier function of gut epithelium. This deleterious effect was prevented in the HFD-HE group, where values similar to those found in the ND group were reached. In the case of gut microbiota composition, improved species richness and diversity were found in both *Nitzschia laevis*-supplemented groups when compared to the ND and HFD groups. At the phylum level, the decreased *Firmicutes*/*Bacteroidetes* ratio values appreciated in the HFD group was reversed in the two groups supplemented with the microalga, reaching values similar to those observed in the ND group. 

Based on the results obtained, the authors concluded that *Nitzschia laevis* supplementation could be an effective tool in the prevention of body weight induced by HFD feeding in mice. In this regard, the beneficial effects induced by *Nitzschia laevis* supplementation in gut epithelium integrity and gut microbiota modulation are highlighted as potential underlying mechanisms. 

## 4. Human Studies

Studies addressed in humans devoted to analyzing the anti-obesity effects of microalgae are scarce to date, in comparison to those carried out using in vitro and in vivo experimental models (Table 3). In humans, Spirulina has been used as a dietary supplement for ameliorating a variety of diseases. In this line, Hernández-Lepe et al. [17] carried out a randomized double-blind crossover controlled clinical trial to evaluate the effect of short-term *Spirulina maxima* supplementation on plasma lipid levels and BMI. For this purpose, young (26 ± 5 years) sedentary men with BMI ≥ 25 kg/m^2^, some of whom suffering from dyslipidemia, were divided into two intervention groups: the Sm group received a supplement of *Spirulina maxima* (4.5 g/day) and the control group received placebo. 

The results showed a significant decrease in TC and TG along with a significant increase in HDL-cholesterol in the Sm group after treatment, when compared with the basal levels. These changes were observed only among dyslipidemic subjects. However, LDL-cholesterol showed no change. In addition, the authors compared the variation of each parameter between both experimental groups and observed that plasma TC level decreased significantly in obese subjects in the Sm treatment, and LDL-cholesterol was lower in overweight, obese, and dyslipidemic subjects enrolled in the Sm treatment, when compared to those in the placebo group. By contrast, TG and HDL-cholesterol levels were not modified. As far as BMI is concerned, a significant reduction was only observed in obese and dyslipidemic subjects after treatment. These results suggest that *Spirulina maxima* supplementation results in a partial improvement of blood lipid profile and BMI in men with excess body weight and dyslipidemia. 

Using the same microalga, Szulinska et al. [18] carried out a randomized, double-blind, placebo-controlled trial addressed on 25–60 year old individuals with obesity (BMI ≥ 30 kg/m^2^), with well-controlled hypertension and without other comorbidities. Participants were divided into two experimental groups: placebo group (four capsules per day of microcrystalline cellulose over 3 months) and spirulina group (four capsules per day of Hawaiian Spirulina over 3 months). Each spirulina capsule contained 0.5 g of *Spirulina maxima*. At the end of the experimental period, spirulina group showed lower BMI, waist circumference, serum TC, LDL-cholesterol, glucose and insulin and total antioxidant state than the placebo group. No differences in serum HDL-cholesterol and TG were observed between groups. 

In another randomized doubled-blind, placebo-controlled trial conducted by Zeinalian et al. [19], the effect of *Spirulina platensis* supplementation on BMI, serum lipids, appetite, and serum vascular endothelial growth factor (VEGF) was studied. Individuals with obesity were divided into two groups, the placebo group and the group that received *Spirulina platensis* twice daily (500 mg each dose). After 12 weeks of intervention, a decrease in body weight, and thus in BMI, was observed, along with a reduction appetite in the group treated with *Spirulina platensis*. With regard to serum lipids, the only change was a significant reduction in TC, while LDL-cholesterol and TG remained unchanged after the intervention. Despite a significant increase in HDL-cholesterol in both treated and placebo groups at the end of the experimental period, there was no change in the mean differences between the two groups. VEGF is an important angiogenic factor implicated in normal and pathological vessel formation that can be an important biomarker of obesity and obesity-related cancer progression. In this study, VEGF remained unchanged after treatment with *Spirulina platensis*. The authors concluded that a dose of 1 g/day of *Spirulina platensis* for 12 weeks had beneficial effects modulating body weight and appetite, while it only modified the serum lipid profile partially. 

*Spirulina platensis* was also used in a randomized, double-blinded, placebo-controlled clinical trial reported by Yousefi et al. [20]. Obese or overweight subjects (BMI: 25–40 kg/m^2^) were distributed into two groups, a placebo and a *Spirulina platensis*-treated group, who followed a restricted calorie diet for 12 weeks. The microalga was administered in four tablets of 500 mg/capsule daily. At the end of the intervention, body weight and waist circumference were reduced in the microalga-supplemented group compared to the control group. Moreover, in this group body fat reduction was higher than that observed in the placebo group. Regarding plasma parameters, TG, LDL-cholesterol, and the LDL/HDL ratio were reduced at the end of the treatment period compared with the baseline in the microalga-treated group. Based on these results, the authors suggested that *Spirulina platensis* could be a useful as a complementary therapy to reduce weight and TG levels.

## 5. Concluding Remarks

Data reported in the literature, and gathered in the present review, show that there is scientific evidence supporting the anti-obesity effect of several microalgae: *Euglena gracilis, Phaeodactylum tricornutum, Spirulina maxima, Spirulina platensis*, and *Nitzschia laevis.* With the exception of one study, the published works carried out in animal models have addressed the effects of microalgae in animals submitted to an obesogenic feeding pattern. Consequently, the results have shown the ability of microalgae to total or partially prevent obesity development associated to this dietary pattern. 

Preclinical studies have revealed some of the mechanisms of action underlying this effect. Depending on the species and concentration, microalgae can inhibit pre-adipocyte differentiation, thus reducing the number of mature adipocytes ready to accumulate TG. Moreover, they reduce de novo lipogenesis and TG assembly, thus limiting the amount of TG to be stored. An increase in lipolysis and fatty acid oxidation can also be observed. Finally, microalgae can induce an increase in energy expenditure via thermogenesis activation in brown adipose tissue, as well as by inducing browning in white adipose tissue. It could be thought that a potential toxic effect of some constituent common in microalgae could be responsible, at least in part, for the reduced lipid retention and weight reduction. However, this possibility can be discarded because in vitro studies have shown no cytotoxicity of microalgae extracts in a wide range of doses. In parallel with the reduction in body fat accumulation, other features which are typical of individuals with obesity, such as enhanced plasma lipid levels, insulin resistance or diabetes, and low-grade inflammation, are also improved by microalgae treatment.

The anti-obesity effect of microalgae, as well as the improvement of several comorbidities observed in preclinical studies, has been confirmed in clinical trials. In this case, due to the experimental design characteristics, the role of microalgae in obesity treatment, rather than in obesity prevention, has been evidenced. 

Concerning the limitations of the reported studies, it should be pointed out that more research is needed to determine which bioactive compounds, present in microalgae, are responsible for their anti-obesity effects, as well as to look for potential synergies among them. In addition, although several mechanisms have been proposed to explain the anti-obesity effects of microalgae, further studies are needed in order to gain more insight concerning this issue. For instance, in several studies increased expression of genes related to thermogenesis has been found, suggesting the activation of this process, but additional studies are needed to confirm that in fact thermogenesis, and consequently energy expenditure, are increased.

## Figures and Tables

**Table 1:** Effects of microalgae in pre-adipocytes and mature adipocytes.

Reference Numbers	Cell Line	Microalgae and Doses	Experimental Design	Effects	Mechanisms
[8]	Human adipose-derived stem cells	*Euglena gracilis Z* extract (5%,10%, or 20%)	Cells were treated during differentiation (7 days) and adipocyte maturation (7 additional days)	At 10% and 20%: ↓ lipid content 44% and 74% respectively At 20%: ↓ Adipogenesis	At 20%: ↓ C/EBPα and PPAR*γ* gene and protein expressions ↓ *Creb*, *Srebp1c*, *C/ebpβ* and *C/ebpδ* gene expression ↓ *Fabp4* and *Lpl* gene expression
[9]	3T3-L1 pre-adipocytes	*Spirulina maxima* extract (50 and 100 µg/mL)	Cells were treated on day 0, 2, 4, and 6 of differentiation (cell harvesting on day 8)	At 100 µg/mL: ↓ Adipogenesis (dose dependent)	At 100 µg/mL: ↓ FAS and C/EBPα protein expression At 50 and 100 µg/mL: ↓ PPARγ and aP2 protein expression ↓ SREBP1c, ACC, LPAATβ, lipin1, and DGAT-1 protein expression
	C3H10T1/2 mesenchymal stem cells	*Spirulina maxima* extract (100 µg/mL)	Cells were treated on day 0, 2, 4, and 6 of differentiation (cell harvesting on day 8)	↓ Adipogenesis	↓ C/EBPα, PPARγ, and aP2 protein expression ↓ SREBP1c, FAS, ACC, LPAATβ, lipin1 and DGAT-1 protein expression
[10]	3T3-L1 pre-adipocytes	*Phaeodactylum tricornutum* extract (250 and 400 µg/mL)	Cells were treated during differentiation (6 days)	At 250 µg/mL ↓ Lipid accumulation At 400 µg/mL ↓ Adipogenesis	At 400 µg/mL ↓ PPARγ and ↑ UCP1 protein expression
[11]	3T3-L1 pre-adipocytes	*Phaeodactylum tricornutum extract* (100 mg/L)	Cells were treated on day 7 of differentiation for 24 h (cell harvesting on day 8)	No differences in lipid content or cytotoxicity	↑ *Cd36* and *Cpt1* gene expression

Special note: Doted lines incorporated for separation between different studies. ACC: Acetyl-CoA carboxylase, AP2: fatty acid binding protein, C/EBP: CCAAT-enhancer-binding protein, CD36: cluster of differentiation 36, CPT1: carnitine palmitoyltransferase 1, CREB: cAMP regulatory element-binding protein; DGAT-1: diacylglycerol O-acyltransferase, FABP4: fatty acid-binding protein 4, FAS: fatty acid synthase, LPAATβ: lysophosphatidic acid acyltransferase β, LPL: lipoprotein lipase, PPARγ: peroxisome proliferator activated receptor γ, SREBP1c: sterol regulatory element-binding protein 1c, UCP: uncoupling protein. ↑ significant increase, ↓: significant decrease.

**Table 2:** Effects of microalgae in animal studies.

Reference Numbers	Animal Model	Microalgae and Doses	Experimental Design	Effects	Mechanisms
[9]	Male ICR mice (4 weeks old)	*Spirulina maxima* extract (SM70EE; 150 and 450 mg/kg BW/day) 6 weeks	HFD: high-fat diet (60% of energy from fat) SM150: HFD + SM70EE 150 mg/kg BW/day SM450: HFD + SM70EE 450 mg/kg BW/day	SM450 group: ↑ Serum HDL-c SM150 and SM450 groups: ↓ Final body weight ↓ Body weight gain ↓ Subcutaneous and abdominal WAT ↓ Serum TG, TC, LDL-c and glucose levels ↓ Adipogenesis in WAT	SM450 group: ↑ pAMPK, PRDM16 and PGC1α protein expression in WAT ↑ UCP1 protein expression in BAT SM150 and SM450 groups: ↓ C/EBPα, PPARγ, and aP2 protein expression in WAT ↑ UCP1 protein expression in WAT ↑ PRDM16 protein expression in BAT
[13]	Male Sprague-Dawley rats (5 weeks old)	*Spirulina maxima* (62.5, 125, and 250 mg/kg BW/day) 4 weeks	LFD: low-fat diet (10% of energy from fat) HFD: high-fat diet (60% of energy from fat) SM 62.5: HFD + 62.5 mg/kg BW/day SM 125: HFD + 125 mg/kg BW/day SM 250: HFD + 250 mg/kg BW/day of	SM 62.5, 125, and 250 SM groups: ↓ Epididymal adipocyte size ↑ Brown adipose tissue index ↑ Serum adiponectin ↓ Serum TNF-α ↓ HOMA-IR ↑ Serum HDL-c/TC ↓ Serum ALT SM 125 and SM 250 groups: ↓ Body weight gain ↑ Brown adipose tissue ↓ Serum leptin ↓ Serum insulin ↓ Serum TC SM 250 group: ↓ Epididymal adipose tissue index ↓ Serum glucose	SM 125 and SM 250 groups: ↓ FAS protein expression (epididymal AT) ↓ *Fasn* gene expression (epididymal AT) ↑ *AdipoR* gene expression (epididymal AT) ↑ pAMPK protein expression (epididymal AT and skeletal muscle) ↑ *AdipoR1*, *Cpt1* and *Ucp2* gene expression (skeletal muscle) ↓ *Srebf1* and *Nfκβ* gene expression (epididymal AT) SM 250 group: ↑ *Atgl* and *Cpt1* gene expression (epididymal AT) The dose is not specified: ↑ pACC, AdipoR1, NAMPT, and SIRT1 protein expression (epididymal AT) ↓ SREBP1 and FAS protein expression (epididymal AT) ↑ AdipoR1, NAMPT and SIRT1 protein expression (skeletal muscle)
[11]	Male C75BL/6J mice (6–8 weeks old)	*Phaeodactylum tricornutum* (PE) extract (100 and 300 mg/kg BW/day) 26 days	HFD: high-fat diet 45% of energy from fat) PE100: HFD + PE 100 mg/kg BW/day PE300: HFD + PE 300 mg/kg BW/day	PE300 group: ↓ Final body weight ↓ Total body fat mass ↓ Epididymal and inguinal tissue ↓ Adiposity index ↑ Energy expenditure PE100 and PE300 group: ↑ % of small adipocytes in inguinal WAT	PE100 group: ↑ *Cd36* and *Ppargc1a* gene expression in BAT PE300 group: ↓ *Lipe*, *Plin1*, and *Lpl* gene expression in epididymal WAT ↑ *Ucp1* and *Cpt* gene expression in inguinal WAT ↑ BAT activation PE100 and PE300 group: ↓ *Lipe* and *Fasn* gene expression in BAT ↑ UCP1 protein expression in BAT
[10]	Female C57BL/6J mice (8 weeks old)	*Phaeodactylum tricornutum* (PE) extract (0.81, 1.62, and 3.25 mg/kg BW/day) 6 weeks	HFD: High-fat diet PE-L: HFD + PE 0.81 mg/kg BW/day PE-M: HFD + PE 1.62 mg/kg BW/day PE-H: HFD + PE 3.25 mg/kg BW/day	PE-H group: ↓ Plasma LDL-c PE-M group: ↓ Plasma TG PE-M and PE-H groups: ↓ Subcutaneous fat volume PE-L, PE-M and PE-H groups: ↓ Body weight gain ↓ inguinal fat depots ↓ Total and abdominal fat volume	PE-H group: ↓ C/EBPα protein expression in WAT PE-M and PE-H groups: ↑ UCP1 protein expression in WAT PE-L, PE-M, and PE-H groups: PPARγ protein expression in WAT
[14]	Male C57BL/6J mice (10 weeks old)	*Euglena gracilis* extract (1%) 8 weeks	Standard diet Standard diet + *Euglena gracilis* extract	↓ Total, perinephric, and epididymal adipose tissue	*↑Lipe* gene expression in epididymal WAT
[15]	Male C57BL/6J mice (10 weeks old)	*Nitzschia laevis* extract (10 or 50 mg/kg BW/day) 8 weeks	HFD (24% of energy from fat) HFD+ 10 mg/kg BW/day of *Nitzschia laevis* extract HFD+ 50 mg/kg BW/day of *Nitzschia laevis* extract	50 mg/kg BW/day: ↓ Body weight ↓ Liver weight and lipid accumulation Improved gut epithelium integrity 10 and 50 mg/kg BW/day: ↓ Epididymal adipose tissue weight and adipocyte diameter ↑ BAT adipocyte number Improved gut microbiota richness and diversity ↑ *Firmicutes/Bacteroidetes* ratio	10 mg/kg BW/day: ↑ *Pgc-1α* gene expression in BAT 50 mg/kg BW/day: ↑ *Occludin* gene expression 10 and 50 mg/kg BW/day: ↑ *Ucp1* gene expression in BAT

Special note: Doted lines incorporated for separation between different studies. ACC: Acetyl-CoA carboxylase, AdipoR1: adiponectin receptor 1, ALT: alanine aminotransferase, AMPK: AMP-activated protein kinase, AP2: fatty acid binding protein, AT: adipose tissue, ATGL: adipose triglyceride lipase, BAT: brown adipose tissue, BW: body weight, CD36: cluster of differentiation 36, C/EBPα: CCAAT-enhancer-binding protein α, CPT: carnitine palmitoyltransferase, FAS: fatty acid synthase, Fasn: fatty acid synthase (gene), HDL-c: high-density lipoprotein cholesterol, HFD: high-fat diet, HOMA-IR: Homeostatic Model Assessment of Insulin Resistance, ICR: Institute of Cancer Research, IR: insulin resistance, LDL-c: low-density lipoprotein cholesterol, LIPE: hormone sensitive lipase, LPL: lipoprotein lipase, NAMPT: nicotinamide phosphoribosyltransferase, NFκB: nuclear factor kB, PGC-1α: peroxisome proliferator-activated receptor gamma co-activator 1α, PLIN1: perilipin 1, PPARγ: peroxisome proliferator activated receptor γ, PPARGC1a: PPARG coactivator 1 alpha, PRDM16: PR domain-containing16, SIRT1: sirtuin 1, SREBP1: sterol regulatory element-binding protein 1, TC: total cholesterol, TG: triglycerides, TNF-α: tumor necrosis factor α, UCP1: uncoupling protein 1, WAT: white adipose tissue. ↑ significant increase, ↓: significant decrease.

**Table 3:** Effects of microalgae in clinical studies.

Reference Numbers	Participants	Microalgae and Doses	Experimental Design	Effects
[17]	52 sedentary young men (26 ± 5 years) with BMI ≥ 25 kg/m^2^ 27 subjects with overweight and 25 subjects with obesity	*Arthrospira (Spirulina) maxima* 4.5 g/day	Two intervention groups: Sm: *S. maxima* supplementation C: Control (Placebo) Duration: 6 weeks + 2 weeks of wash-out + 6 weeks (crossover for the supplementation interventions)	Intra-group (pretreatment vs. post) comparisons (Sm): ↓TC and TG: only in subjects with dyslipidemia. LDL-c: no changes ↑ HDL-c: only in in subjects with dyslipidemia. Inter-group comparisons (Sm vs. C): ↓TC: only in in subjects with obesity. TG: no changes ↓ LDL-c: in in subjects with overweight, obesity or dyslipidemia. HDL-c: no changes ↓ BMI: in subjects with obesity or dyslipidemia.
[18]	50 patients (25 women and 25 men, 25–60 years old) with obesity (BMI ≥ 30 kg/m^2^) and well-controlled hypertension	*Arthrospira platensis* Four-daily dosage of 500 mg each	Two intervention groups: Placebo Spirulina Duration: 12 weeks	Effects vs. placebo: ↓ BMI and waist circumference ↓ Serum TC, LDL-c, glucose, and insulin ↑ Total antioxidant state
[19]	56 individuals with obesity (20–50 years old) with BMI ≥ 30 kg/m^2^	*Arthrospira platensis* Twice-daily dosage of 500 mg each	Two intervention groups: Intervention group: supplementation of *S. platensis* in a dosage of 500 mg twice a day over 12 weeks Control group: two pills of placebo daily over 12 weeks.	↓ BMI (↓ BW) ↓ TC TG: no changes LDL-c: no changes HDL-c: no changes VEGF: no changes ↓ Appetite
[20]	52 subjects with overweight or obesity with BMI between 25 and 40 kg/m^2^	*Spirulina platensis* Four-daily dosage of 500 mg/day	Two intervention groups Intervention SP group: supplementation of SP extract (four tablets per day) for 12 weeks with a restricted calorie diet Placebo group: placebo extract (four tablets per day) for 12 weeks with a restricted calorie diet	↓ Body weight ↓ Waist circumference ↑ Body fat reduction ↓ Plasma TG, LDL-c ↓ LDL-c/HDL-c ratio

Special note: Doted lines incorporated for separation between different studies. BMI: body mass index; BW: body weight; C: control (Placebo); HDL-c: high density lipoprotein -cholesterol; LDL-c: low density lipoprotein-cholesterol; Sm: *Spirulina* without exercise; TC: total cholesterol; TG: triglycerides; VEGF: vascular endothelial growth factor. ↑ significant increase, ↓: significant decrease.

## Abbreviations

ACC	acyl-CoA carboxylase
AdipoR1	adiponectin receptor
ALT	alanine aminotransferase
Ap2	fatty acid binding protein
ATGL	adipose triglyceride lipase
AUC	area under the curve
BAT	brown adipose tissue
BMI	body mass index
CLS	crown-like structures
C/EBPα	CCAAT-enhancer-binding protein α
CD36	cluster of differentiation 36
CPT1a	carnitine palmitoyltransferase 1a
CREB	cAMP regulatory element-binding protein
DGAT1	diacylglycerol acyltransferase-1
EPA	eicosapentanoic acid
FABP4	fatty acid binding protein 4
FAS	fatty acid synthase
G6PDH	glucose-6-phosphate dehydrogenase
hASCs	human adipose-derived stem cells
HDL	high-density lipoprotein
HFD	high-fat diet
HOMA-IR	Homeostatic Model Assessment for Insulin Resistance
HSL	hormone sensitive lipase
LDL	low-density lipoprotein
LPAATβ	lysophosphatidic acid acyltransferase β
LPL	lipoprotein lipase
MAC-2	macrophage marker galectin-3
ME	malic enzyme
MEST	mesoderm-specific transcript homolog protein
NAMPT	nicotinamide phosphoribosyltransferase
ND	normal diet
NFκΒ	nuclear factor κΒ
pAMPK	5’ AMP-activated protein kinase
Plin1	perilipin 1
PGC1α	peroxisome proliferator-activated receptor gamma coactivator 1-alpha
PPAR γ	peroxisome proliferator-activated receptor γ
PRDM16	PR domain containing 16
SIRT1	sirtuin 1
SREBP1c	sterol regulatory element-binding transcription factor 1c
TC	total cholesterol
TG	triglyceride
UCP1	uncoupling protein 1
VEGF	vascular endothelial growth factor
WAT	white adipose tissue
